# Animal Models of Steatosis (NAFLD) and Steatohepatitis (NASH) Exhibit Hepatic Lobe-Specific Gelatinases Activity and Oxidative Stress

**DOI:** 10.1155/2019/5413461

**Published:** 2019-02-03

**Authors:** Giuseppina Palladini, Laura G. Di Pasqua, Clarissa Berardo, Veronica Siciliano, Plinio Richelmi, Stefano Perlini, Andrea Ferrigno, Mariapia Vairetti

**Affiliations:** ^1^Department of Internal Medicine and Therapeutics, University of Pavia, Pavia, Italy; ^2^Fondazione IRCCS Policlinico S. Matteo, University of Pavia, Pavia, Italy

## Abstract

Animal models of obstructive cholestasis and ischemia/reperfusion damage have revealed the functional heterogeneity of liver lobes. This study evaluates this heterogeneity in nonalcoholic fatty liver disease (NAFLD) and nonalcoholic steatohepatitis (NASH) rat models. Twelve-week-old Obese and Lean male Zucker rats were used for NAFLD. Eight-week-old male Wistar rats fed with 8-week methionine-choline-deficient (MCD) diet and relative control diet were used for NASH. Gelatinase (MMP-2; MMP-9) activity and protein levels, tissue inhibitors of metalloproteinase (TIMPs), reactive oxygen species (ROS), and thiobarbituric acid-reactive substances (TBARS) were evaluated in the left (LL), median (ML), and right liver (RL) lobes. Serum hepatic enzymes and TNF-alpha were assessed. An increase in gelatinase activity in the NASH model occurred in RL compared with ML. TIMP-1 and TIMP-2 displayed the same trend in RL as ML and LL. Control diet RL showed higher MMP-9 activity compared with ML and LL. No significant lobar differences in MMP-2 activity were detected in the NAFLD model. MMP-9 activity was not detectable in Zucker rats. TIMP-1 was lower in LL when compared with ML while no lobar differences were detectable for TIMP-2 in either Obese or Lean Zucker rats. Control diet rats exhibited higher ROS formation in LL versus RL. Significant increases in TBARS levels were observed in LL versus ML and RL in control and MCD rats. The same trend for ROS and TBARS was found in Obese and Lean Zucker rats. An increased serum TNF-alpha occurred in MCD rats. A lobar difference was detected for MMPs, TIMPs, ROS, and TBARS in both MCD and Zucker rats. Higher MMP activation in RL and higher oxidative stress in the LL, compared with the other lobes studied, supports growing evidence for functional heterogeneity among the liver lobes occurring certainly in both NAFLD and NASH rats.

## 1. Introduction

Among emergent metabolic chronic liver diseases, nonalcoholic fatty liver disease (NAFLD) and its more advanced form, nonalcoholic steatohepatitis (NASH), are becoming a major public health problem in industrialized countries [1, 2]. The estimated worldwide prevalence is 4-46% for NAFLD and 3%-5% for NASH [3]. The highest prevalence of NAFLD is observed in Western countries (17% to 46%) where it is poised to become the most important cause of morbidity and mortality for chronic liver disease [2, 4].

Animal models are an essential tool for the identification of the mechanisms driving the pathogenesis and progression of NAFLD to NASH. Ideally, experimental models should reflect the etiology, disease progression, and pathology of human NAFLD. Unfortunately, currently available models, MCD diet, Western diet, and high-fat diet, are complementary and each of them partially reflects the real picture of human NAFLD [5]. The available experimental models can be classified into genetic and nutritional: the main genetic model is Zucker rat (fa/fa), a genetic model of metabolic syndrome with obesity, while the most commonly used nutritional model employs a methionine- and choline-deficient diet (MCD diet) [5]. It is a very reproducible model, consistently inducing a phenotype of severe NASH after 8 weeks of administration [6].

The liver parenchyma displays a functional organization known as metabolic zonation: the hepatocytes lined up between the sinusoids along the porto-central axis show structural and functional heterogeneity [7]. However, in addition, there is increasing evidence of functional heterogeneity in the individual liver lobes, revealing an unexplained interlobular variability as shown by heterogeneous damage distribution when different lobes are compared [8]. Many differences between liver lobes are found in several hepatic diseases and toxic injury such as chemical carcinogenesis, cirrhosis, and acetaminophen toxicity [9–11]. We previously demonstrated that a functional lobar heterogeneity of the liver exists in ischemia/reperfusion and obstructive cholestasis animal models, indicating that different events such as modulation of the extracellular matrix (ECM) and oxidative stress occur with different intensities in the hepatic lobes [12, 13].

The goal of the present study was to investigate presumed liver lobe heterogeneity in nonalcoholic fatty liver disease (NAFLD) and nonalcoholic steatohepatitis (NASH) models, in terms of alteration of the ECM, matrix metalloproteinase (MMP) activity, and specific inhibitors (TIMPs) and of oxidative stress content, ROS, and TBARS formation.

## 2. Material and Methods

### 2.1. Animals

**Figure 1:**
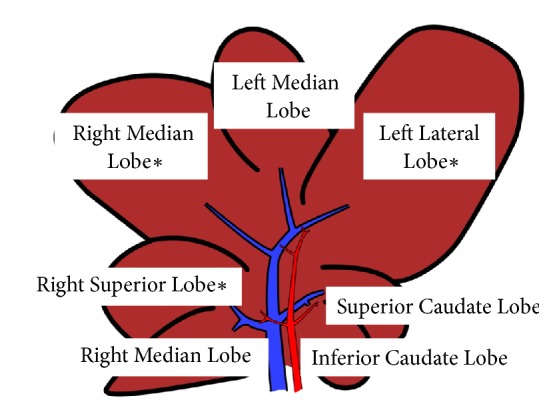
Graphic (schematic) representation of hepatic lobes. Liver samples were collected (**∗**) from superior right lobe (RL), right median lobe (ML), and lateral left lobe (LL).

Zucker rats represent a well-characterized model of NAFLD. Fourteen 11-week-old male obese (fa/fa) Zucker rats and age-matched lean (fa/-) were used. Animals (n=7 each group) were supplied by Charles River, Italy. The most widely used diet to induce NASH is the methionine-choline-deficient (MCD) diet. Fourteen 8-week-old male Wistar rats were fed with MCD diet (Laboratorio Dottori Piccioni, Milano, Italy), or with an isocaloric diet supplemented by choline and methionine (Control) for 8 weeks. Animals (n=7 each group) were supplied by Charles River, Italy. Animal models used were approved by the Italian Ministry of Health and by the local University Animal Care Commission (Document number 2/2012). At the time of sacrifice, on the basis of rat lobar structure, recently described by Sanger et al. [14], liver samples from the superior right lobe (RL), right median lobe (ML), and lateral left lobe (LL) were collected and snap frozen in liquid nitrogen (Figure 1); serum blood samples were also collected.

### 2.2. Assays

Liver injury was assessed by serum level evaluation of alanine transaminase (ALT) and aspartate transaminase (AST) using a commercial kit (Sigma). Serum levels of TNF-alpha were evaluated by a commercial ELISA kit according to the manufacturing procedures (R&D Systems, Minneapolis, MN). Determination of hepatic reactive oxygen species (ROS) was followed by the conversion of 2′,7′-dichlorofluorescein diacetate (H2DCFDA) to fluorescent 2′,7′-dichlorofluorescein (DCF) as previously described [15]. The extent of lipid peroxidation in terms of thiobarbituric acid-reactive substances (TBARS) formation was measured as previously described [16].

### 2.3. Tissue Sources and Hepatic Protein Isolation

After sacrifice, hepatic lobes were quickly excised and placed in cold (4°C) buffer (30 mM histidine, 250mM sucrose, 2 mM EDTA, pH 7.2) to remove blood. Liver was weighed and subsequently cut, frozen in liquid nitrogen and stored at -80°C, until use. Hepatic protein was extracted by homogenisation (IKA-Ultraturrax T10) of frozen liver tissue, in an ice-cold extraction buffer (1:10 wt/vol) containing 1% Triton X-100, 500 mmol/L Tris-HCl, 200 mmol/L NaCl, and 10mmol/L CaCl2, pH 7.6 [17]. The homogenate was then centrifuged (30 min. at 12.000 rpm at 4°C) and the protein concentration of the supernatant was measured with the colorimetric Lowry method [18]. Samples were stored at -20°C before use. MMP-2 (gelatinase A; EC 3.4.24.24), MMP-9 (gelatinase B; EC 3.4.24.35), TIMP-1, and TIMP-2 protein levels were determined with a commercial ELISA kit (Abnova).

### 2.4. MMP-2 and MMP-9 Zymography

In order to detect MMPs lytic activity, the hepatic extracts were normalized to a final concentration of 400 *μ*g/mL in sample loading buffer (0.25 M Tris-HCl, 4% sucrose wt/vol, 10% SDS wt/vol and 0.1% bromphenol blue wt/vol, pH 6.8). After dilution the samples were loaded onto electrophoretic gels (SDS-PAGE) containing 1 mg/mL of gelatin under nonreducing conditions [19] followed by zymography as described previously [20]. The zymograms were analyzed by densitometer (GS900 Densitomer; BIORAD, Hercules, CA, USA) and data were expressed as optical density (OD), related to 1 mg/mL protein content.

### 2.5. Statistical Analysis

Results are expressed as mean ± standard error. Comparisons between groups were performed by unpaired t test. When data distribution was not normal according to the Kolgonorov-Smrna test, a Mann-Witney test was used. All statistical procedures were performed using the MedCalc statistical software package (11.6.0.0 version). A value of p<0.05 was considered significant.

## 3. Results

### 3.1. NASH and Lobe-Specific Levels of MMPs and TIMPs

**Figure 2:**
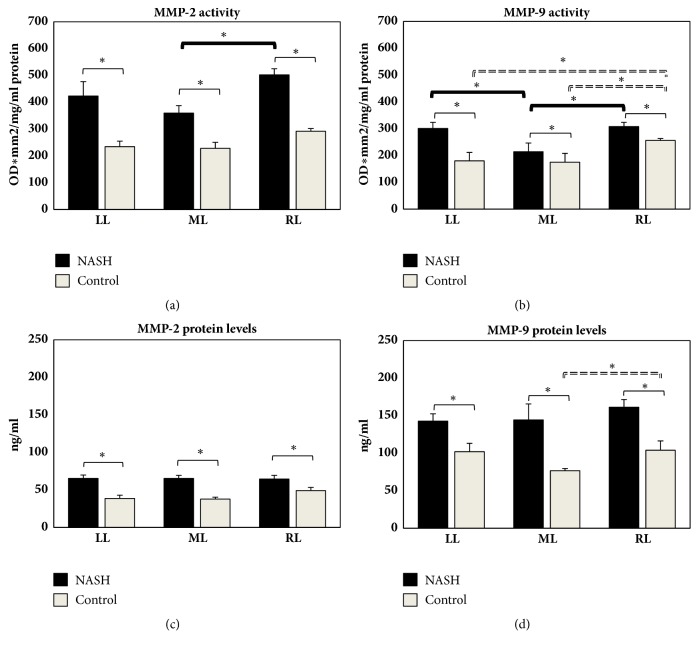
Hepatic content in MMP-2 and MMP-9 activity ((a) and (b)) and MMP-2 and MMP-9 protein levels ((c) and (d)) obtained from LL, ML, and RL in NASH and control rats. MMP gelatinase activity is expressed as optical density (OD) for mm^2^, related to 1 mg/mL protein content. MMP protein content is expressed in ng/mL. Data are shown as mean values ± SE. *∗*p<0.05.

A general increase in gelatinolytic activity was observed in the NASH model, in the RL. In particular, gelatin zymography revealed a statistical difference between the liver lobes: MMP-2 and MMP-9 activity was significantly increased in the RL compared with the ML in the MCD rats (Figures 2(a) and 2(b)). MMP-9 activity was lower in the ML when compared with the LL (Figure 2(b)). Although not significant, a similar trend occurred for MMP-2 in the control livers (Figure 2(a)). A marked increase in MMP-9 activity was also found in the RL when compared with the ML and LL in control rats (Figure 2(b)). A significant increase in MMP-2 and MMP-9 activity was found in the RL, ML, and LL of NASH animals compared to the respective control animals (Figures 2(a) and 2(b)).

The analysis of MMP protein levels revealed comparable MMP-2 levels between lobes in the control and MCD animals (Figure 2(c)). A mild increase in MMP-9 occurred in the RL in MCD and reached significantly different levels in the control animals when compared with the ML (Figure 2(d)). Higher MMP-2 and MMP-9 protein levels in the RL, ML, and LL of NASH rats compared to their respective control animals were also found (Figures 2(c) and 2(d)).

**Figure 3:**
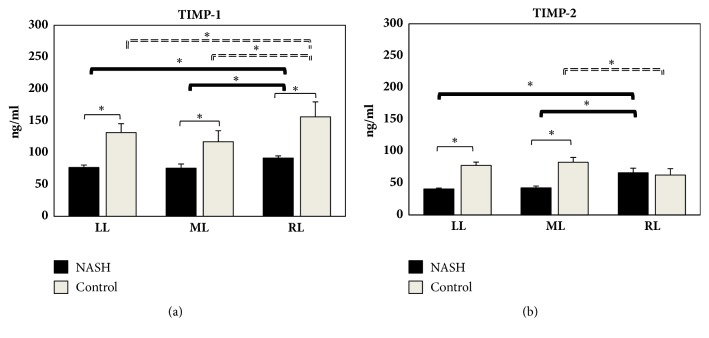
Hepatic content in TIMP-1 (a) and TIMP-2 (b) obtained from LL, ML, and RL in MCD and control rats. TIMP levels are expressed in ng/mL. Data are shown as mean values ± SE. *∗*p<0.05.

TIMP-1 and TIMP-2 levels were higher in the RL in the NASH model, when compared with the ML and LL (Figure 3(a)). The same trend occurred in the control animals for TIMP-1. A lower level of TIMP-2 in NASH rats was found in the RL when compared with the ML. This trend occurred for the ML versus RL in the control animals (Figure 3(b)). Lower TIMP-1 levels in the RL, ML, and LL in NASH rats compared to their respective control animals were also found (Figure 3(a)). The same trend occurred for TIMP-2 in the LL and ML (Figure 3(b)).

### 3.2. NAFLD and Lobe-Specific Levels of MMPs and TIMPs

**Figure 4:**
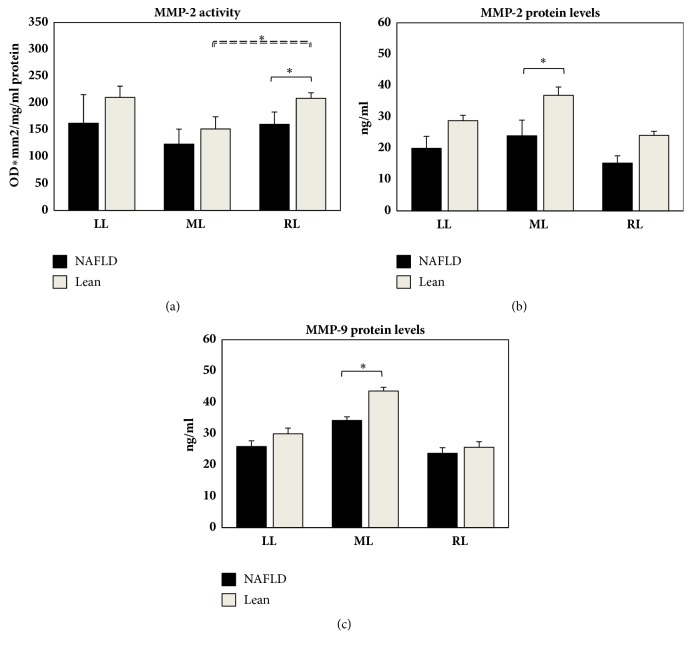
Hepatic content in MMP-2 activity (a) and MMP-2 and MMP-9 protein levels ((b) and (c)) obtained from LL, ML, and RL in NAFLD and Lean rats. MMP gelatinase activity is expressed as optical density (OD) for mm2, related to 1 mg/mL protein content. MMP content is expressed in ng/mL. Data are shown as mean values ± SE. *∗*p<0.05.

The evaluation of MMP-2 in the NAFLD animals revealed low levels in the ML, though not significantly whereas this activity was significant in Lean Zucker rats (Figure 4(a)). No MMP-9 activity was detectable in Obese and Lean Zucker rats. Lower levels of MMP-2 activity were found in the RL of NAFLD rats compared to the respective Lean animals (Figure 4(a)).

The analysis of MMP protein levels in NAFLD animals and their control animals showed a slight decrease in MMP-2 and MMP-9 protein content in the RL when compared with the ML (Figures 4(b) and 4(c)). Although significant only for the ML, lower levels of MMP protein levels were found in NAFLD rats compared to the ML in Lean animals (Figures 4(b) and 4(c)).

**Figure 5:**
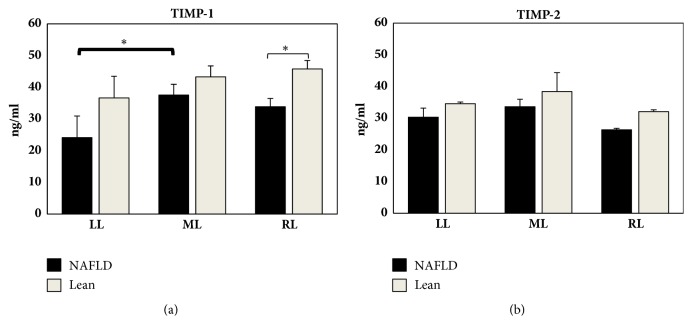
Hepatic content in TIMP-1 (a) and TIMP-2 (b) obtained from LL, ML, and RL in NAFLD and Lean rats. TIMP levels are expressed in ng/mL. Data are shown as mean values ± SE. *∗*p<0.05.

TIMP-1 was significantly higher in the ML when compared with the LL in NAFLD rats (Figure 5(a)). Comparable levels for TIMP-1 were found in lobes from Lean Zucker rats (Figure 5(a)). No difference was detectable for TIMP-2 either in Obese or Lean Zucker rats. Comparable TIMP-1 and TIMP-2 levels in the RL, ML, and LL in Obese Zucker rats compared to Lean animals were found except for TIMP-1 in RL (Figures 5(a) and 5(b)).

### 3.3. Lobe-Specific ROS and TBARS Levels in NASH and NAFLD Models

**Figure 6:**
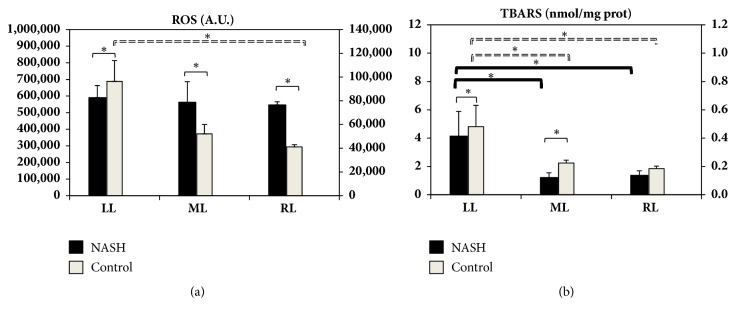
Hepatic levels of ROS (a) and TBARS (b) obtained from LL, ML, and RL in NASH and control rats. Data are shown as mean values ± SE. *∗*p<0.05.

In the NASH model, no difference was detectable between lobes in ROS levels (Figure 6(a)). On the contrary, in control rats, lower ROS content was found in the RL when compared with the LL (Figure 6(a)). Higher hepatic TBARS levels were observed in the LL as compared with the RL and ML in NASH and control animals. A marked increase in TBARS was found when the LL, ML, and RL in NASH rats were compared to their respective control animals (Figure 6(b)).

**Figure 7:**
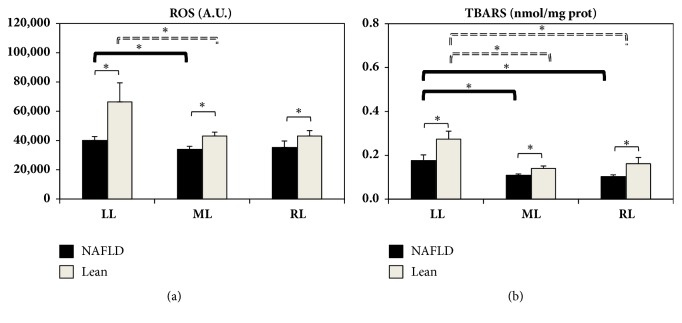
Hepatic levels of ROS (a) and TBARS (b) obtained from LL, ML and RL in NAFLD and Lean rats. Data are shown as mean values ± SE. *∗*p<0.05.

In NAFLD rats, a higher ROS concentration was found in the LL when compared with the ML (Figure 7(a)). The same trend occurred in Lean Zucker rats (Figure 7(a)). Lower levels of ROS were found when the LL, ML, and RL obtained from NAFLD rats were compared with the respective Lean group (Figure 7(a)). Higher TBARS levels were found in the LL in both Obese and Lean and Zucker rats as compared with the respective ML and RL (Figure 7(b)). Lower levels of TBARS were found when the LL, ML, and RL obtained from Obese Zucker rats were compared with the respective lobes of the Lean group (Figure 7(b)).

### 3.4. Liver Injury in NASH and NAFLD Models

Serum AST and ALT increased in NASH animals as compared with the control group (Table 1). The same was also true for TNF-alfa concentration, an index of Kupffer cell activation (Table 1). In Zucker rats, only an increase in ALT was found in the obese animals (Table 1); no difference was detected for the serum TNF-alpha concentration between the Obese and Lean Zucker animals (Table 1). Comparing the NAFLD model with the NASH model, significantly lower TNF-alpha levels were found in the Obese Zucker rats when compared with the MCD rats (Table 1).

## 4. Discussion

### 4.1. Lobe-Specific MMP Activity and TIMP Levels

The rat model used in this study cover the spectrum of liver pathology observed in NASH ranging from hepatic steatosis to inflammation progression to fibrosis. In our study, rats fed the MCD diet for 8 weeks developed steatohepatitis with markers of inflammation. Interestingly, in NASH fibrogenesis, MMPs and TIMPs may play a role not only into the balance between the formation and the degradation of ECM composition [21] but also into the signal transduction for tissue recovery to normal condition [22].

TNF-alpha, an inflammatory cytokine modulating MMPs involved in repair and remodeling, plays a major role in the progression from steatosis to NASH [23]. In the present work, MCD animals, which spontaneously exhibited NASH, showed a marked increase in TNF-alpha associated with upregulated MMPs, both MMP-2 and MMP-9 activity, higher in the RL when compared with the ML and LL. This event also occurred in the RL for MMP-9 in the control rats. The relative increase in fibrosis in the RL than in the LL may be one of the causes of more markedly impaired regenerative capacity of the RL than LL [10]. These results are in line with our previous studies using both control rats and other models of liver disease such as cholestasis and ischemia/reperfusion (I/R) damage: MMP activity was particularly high in the RL as compared with the ML and LL [12, 13].

TIMP-1, a natural inhibitor of MMP-9, is the most relevant TIMP in toxic liver injury and dramatically upregulated by inflammatory cytokines such as TNF-alpha [24]. Here we found higher levels of TIMP-1 in control rats associated with low levels of MMP-9 when compared with NASH rats. In our study we also report that TIMPs and MMPs are concomitantly higher in the RL when compared with the ML and LL. Our results are supported by previous data in which TIMP-1 in steatotic liver grafts were associated with high levels of MMP-9 activity suggesting that MMP-9 expressed in the presence of fat is not completely regulated by TIMP-1 inactivation [25].

Using the genetic model of NAFLD, we also detected lobe-heterogenicity for MMP-2 activity; on the contrary, MMP-9 activity was undetectable, in keeping with the findings of other authors in both liver [25] and kidney [26]. Furthermore, we confirm that the gelatinolytic activity in Obese Zucker rats was lower as compared with Lean rats as already reported in isolated glomeruli [26]. Our data further show that when comparing different lobes heterogeneous distribution of TIMP-1 occurs in a genetic NAFLD model.

### 4.2. Lobe-Specific Oxidative Stress

In the present study we found that the LL exhibits increased oxidative stress that was superimposable in both models considered. High vulnerability to oxidative stress is responsible for the “second hit” in the spontaneous progression from simple steatosis to NASH [27]. In addition to cellular damage by massive membrane lipoperoxidation, as demonstrated by elevated TBARS, ROS can act as second messengers in the control of genes encoding proinflammatory cytokines such as TNF-alpha [5]. The present study also supports the association between high levels of serum TNF-alpha with high levels of hepatic TBARS in NASH rats and the oxidative stress appears particularly elevated in the LL. The absence of lobe-specific concentrations of ROS in NASH animals is probably due to their uncontrolled increase in this model. On the contrary, high ROS levels were detectable in the LL of control rats. Higher ROS content was found in the LL versus the RL and ML although with no increase in serum TNF-alpha and in the presence of low levels of hepatic ROS, in the NAFLD model, too. Hence, our data suggest that differences in ROS formation in the LL were not only associated with the NAFLD or NASH model as it was also found in control animals and Lean Zucker rats. Thus, TBARS analysis was used as a predictive marker in patients affected by NAFLD/NASH [28, 29] because TBARS were associated with the fatty liver stage [30]. In the present study, TBARS levels demonstrated that interlobar differences exist supporting previous data about the existence of specific TBARS content in hepatic samples collected from different lobes as previously observed in livers submitted to I/R or obstructive cholestasis [12, 13].

The lobe-specific heterogeneity could be ascribed to the differential blood supply: recently Sanger et al. described the intrahepatic vascular anatomy in liver rats and mice; of note, the lobar borders of the liver do not always match vascular territorial borders [14]: the ML and the lateral lobe are supplied by the main stem of the portal vein while the right median portal vein (2nd order) supplies the right ML [14]. Furthermore, it might be that a partial mixing of blood from the gastrointestinal tract and spleen occurs leading to difference in delivery of different nutrients or toxins to the liver lobes. This is also known as Portal Streamlining [8]. Recent data reported that after radiopharmaceutical injection a higher uptake in the LL compared to the RL occurred and that this ratio did not correlate with any epidemiological or clinical features [31]. The lobe difference could be ascribed to a phylogenetic difference: the LL is older whereas the RL is more recent as reported by Jacobsonn et al. [32]. Furthermore, a different distribution of vagal afferent neurons in the rat livers suggested that different parts may have a different functional role [33]. Based on the above results, an explanation of lobe heterogeneity may be ascribed to different microcirculations and different innervations associated with a specific response of the various hepatic cell types in which TNF-alpha and ROS are involved in intercellular communication [34].

## 5. Conclusion

**Figure 8:**
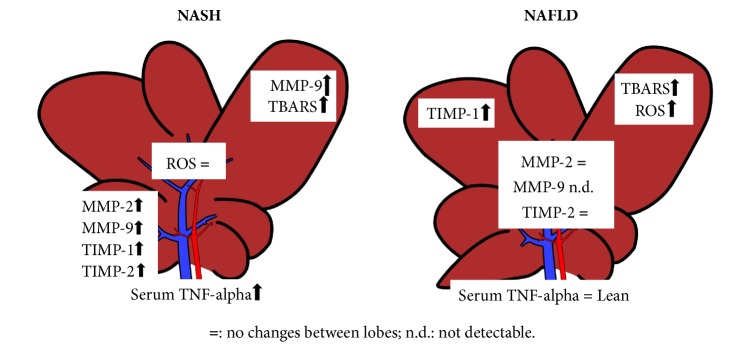
Schematic representation of liver lobes heterogeneity in NASH and NAFLD models (MMPs, matrix metalloproteinases; TIMPs, tissue inhibitors of metalloproteinase ROS, reactive oxygen species; TBARS, thiobarbituric acid-reactive substances).

Whereas intralobular hepatic heterogeneity is extensively described, only a few studies have reported the difference between hepatic lobes. In particular, the present work exhibits, in control rats, lobe-specific heterogeneity in MMPs, TIMPs, and oxidative stress that persists and appears to be amplified during liver injury such as NASH (Figure 8). Although the reason for this different metabolic behavior observed in different lobes is an unsolved mystery, this study supports the growing evidence for functional heterogeneity between the liver lobes already observed in other hepatic diseases, with the same trend, also occurring in NAFLD and NASH.

## Figures and Tables

**Table 1:** Serum enzymes and TNF-alpha in NASH and NAFLD rats.

		ALT	AST	TNF-alpha
		(U/L)	(U/L)	(pg/mL)
**NASH**	*Control *	30.8±2	97.8±2	26.8±2.2
	*MCD *	166.2±23*∗*	245.1±39*∗*	36.7±2.6*∗*^§^

**NAFLD**	*Lean Zucker*	66.2±4.3	112.3±2.8	10.2±0.5
	*Obese Zucker*	114.5±20*∗*	116.1±10	9.5±0.4

*∗*p < 0.05 versus Control. ^§^p < 0.05 versus Obese Zucker. These are the mean results of 7 different experiments ± SE.

## Data Availability

The data used to support the findings of this study are available from the corresponding author upon request.
